# Comparative HIV-1 Proviral Dynamics in Two Individuals That Maintained Viral Replication Control with or without Antiretroviral Therapy following Superinfection

**DOI:** 10.3390/v14122802

**Published:** 2022-12-15

**Authors:** Suwellen Sardinha Dias de Azevedo, Fernanda H. Côrtes, Larissa M. Villela, Brenda Hoagland, Beatriz Grinsztejn, Valdilea G. Veloso, Mariza G. Morgado, Gonzalo Bello

**Affiliations:** 1 Laboratório de AIDS and Imunologia Molecular, Instituto Oswaldo Cruz—FIOCRUZ, Rio de Janeiro 21040-360, Brazil; 2 Instituto Nacional de Infectologia Evandro Chagas-INI, FIOCRUZ, Rio de Janeiro 21040-360, Brazil

**Keywords:** HIV controllers, HIV-superinfection, proviruses, diversity

## Abstract

The analysis of the HIV-1 proviral dynamics after superinfection in the context of both natural and antiretroviral therapy (ART)-mediated suppression could yield unique insights into understanding the persistence of viral variants that seeded the infected cells at different times. In this study, we performed a longitudinal analysis of the env diversity of PBMC-associated HIV DNA quasispecies in two HIV controllers (EEC09 and VC32) that were superinfected with subtype F1 viruses several years after primoinfection with subtype B viruses. Patient EEC09 started ART soon after superinfection, while patient VC32 maintained a natural control of virus replication for at least six years following the superinfection. Our analysis revealed no significant temporal changes in the overall proportion of primo-infecting and superinfecting proviral variants over 2–3 years after superinfection in both HIV controllers. Upon the introduction of ART, individual EEC09 displayed no evidence of HIV-infected cell turnover or viral evolution, while subject VC32 displayed some level of HIV-infected cell reseeding and detectable evolution (divergence) of both viral variants. These results confirm that proviral variants that seeded the reservoir at different times throughout infection could persist for long periods under fully suppressive ART or natural viremic control, but the HIV-1 proviral dynamics could be different in both settings.

## 1. Introduction

People living with HIV (PLWH) need lifelong antiretroviral therapy (ART) to achieve durable control of HIV-1 replication because a latent reservoir of long-lived HIV-infected cells harboring replication-competent proviruses is early established during infection and could be reactivated upon therapy cessation [1,2]. Some rare individuals called HIV controllers (HIC) spontaneously control HIV-1 replication for a long time without ART and are designated as elite controllers (EC) if they suppress plasma viremia to undetectable levels (usually <50 copies/mL) or viremic controllers (VC) if they suppress plasma viremia to low levels (usually 50–2000 copies/mL) [3]. HICs are considered a model of HIV remission or functional cure [4]. Still, it is unclear if the seeding dynamics and longevity of the HIV proviral reservoir observed under the natural control of viremia are comparable to that observed under suppressive ART.

In a previous study, we identified two HICs primo-infected with subtype B viruses and superinfected with subtype F1 viruses several years later [5]. Although both individuals maintained the plasma viremia at low levels after reinfection, the proviruses displayed quite distinct short-term dynamics. The subject EEC09, which was formerly classified as an EC [6], displayed a rapid turnover of the HIV-infected cells and the superinfecting subtype F1 virus rapidly become the dominant proviral variant. By contrast, the subject VC32, which was formerly classified as a VC [6], displayed a slow turnover of the proviruses composition, and the primo-infecting subtype B virus continued to persist as the dominant proviral variant after superinfection. The proviral diversity was previously assessed until the peak viremia, after which subject EEC09 started ART and subject VC32 recovered viremic control while remaining ART-free.

This scenario provides a unique setting to compare the proviral dynamics of both primo-infecting and superinfecting viruses during treated and untreated infections in HICs. To this end, we assess the genetic diversity of the PBMC-associated HIV DNA quasispecies by performing single genome amplification of the env gene over two years of fully suppressive ART (<50 copies/mL) in subject EEC09 and three years of spontaneous control of viremia (50–100 copies/mL) in subject VC32. We next reconstructed the phylogenetic relationships between the HIV DNA env sequences here obtained and those obtained before and immediately after the superinfection to estimate the turnover and divergence of subtype B and F1 variants that seeded the HIV-infected cells at different times.

## 2. Materials and Methods

### 2.1. Study Subjects

**Figure 1:**
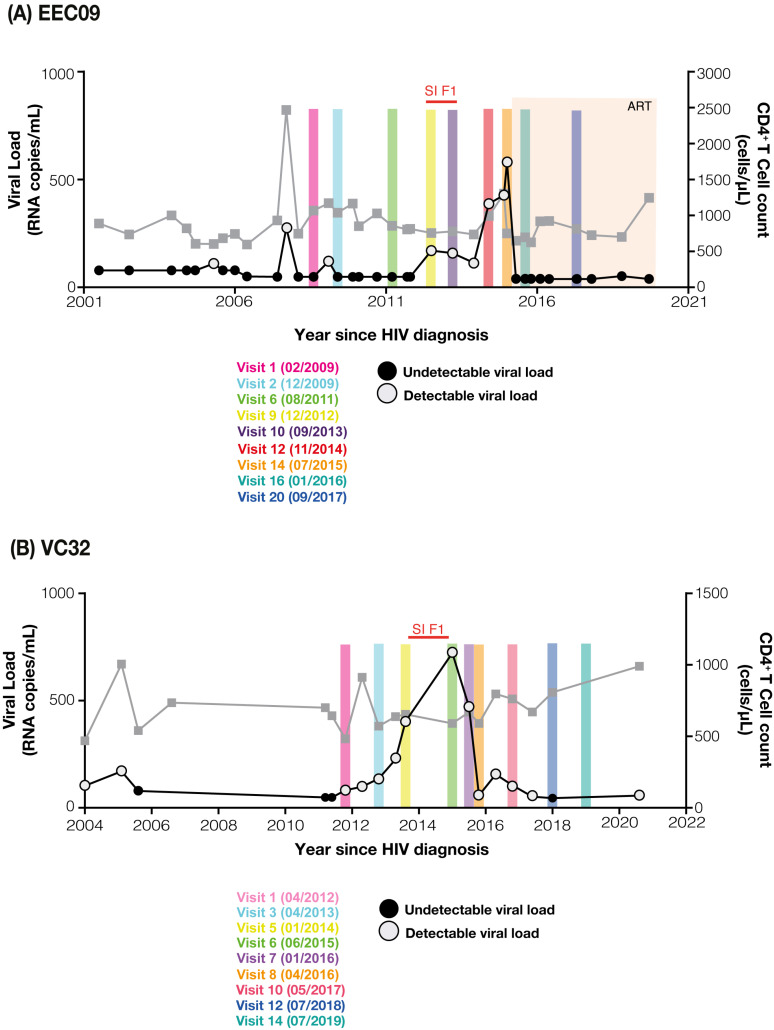
Clinical follow-up from EEC09 (**A**) and VC32 (**B**) individuals. Plasma HIV RNA viral load (copies/mL, circles) and CD4^+^ T cell counts (cells/µL, squares) since HIV diagnosis of individuals EEC09 and VC32 are shown on the left and the right Y-axis, respectively. HIV RNA viral load measurements after the start of combined antiretroviral therapy (ART) are highlighted in the beige frame. The range of superinfection (SI) with the F1 variant of HIV-1 is highlighted in red. Colored shaded areas indicate the time points (visit, month/year) selected for the DNA quasispecies analysis.

Subjects EEC09 and VC32 were followed up at the Instituto Nacional de Infectologia Evandro Chagas (INI) from Rio de Janeiro (Brazil) and provided written informed consent documents approved by the INI Institutional Review Board (Addendum 049/2010) and the Brazilian National Human Research Ethics Committee (CONEP 14430/2011). The main clinical characteristics of the two individuals were described in a previous study [5]. Subject EEC09 is a 52-year-old homosexual male diagnosed with HIV-1 in 2001 and remained treatment-naive until June 2015, when he initiated ART—a triple combination composition consisting of tenofovir (TDF), lamivudine (3TC), and efavirenz (EFV)—after the proposal of the clinicians, following the recent recommendation of the Brazilian Ministry of Health. At enrollment in our cohort in February 2009, subject EEC09 was classified as an EC since most (≥70%) of his plasma VL determinations were below the limit of detection for the available assays (<50–80 copies/mL). VC32 is a 43-year-old homosexual male diagnosed in 2004 and remains antiretroviral-naïve until the present (personal decision). At study entry in April 2012, subject VC32 was classified as a VC since most (≥70%) of his plasma RNA VL determinations were between 80 and 2000 copies/mL. Longitudinal analyzes of the proviral quasispecies of individuals EEC09 (2009-2017) and VC32 (2012–2019) were performed using the HIV-1 env gene sequences generated in the previous study [5] and new sequences generated in this study. For the present study, the proviruses were assessed in two different follow-up visits of subject EEC09 (2016–2017) and three different follow-up visits of subject VC32 (2017–2019 (Figure 1).

#### 2.1.1. CD4^+^ T- Cell Counts and Plasma HIV-1 RNA Quantification

Absolute CD4+ T cell counts were obtained using the MultiTest TruCount-kit and the MultiSet software on a FACSCalibur flow cytometer (BD Biosciences, San Jose, CA, USA). Plasma VL was measured according to the Brazilian Ministry of Health guidelines, with methodologies being updated over time to improve sensitivity: Nuclisens HIV-1 RNA QT assay (Organon Teknika, Durham, NC, USA, limit of detection: 80 copies/mL) from 1999 to 2007; the Versant HIV-1 3.0 RNA assay (bDNA 3.0, Siemens, Tarrytown, NY, USA, limit of detection: 50 copies/mL) from 2007 to 2013; and the Abbott RealTime HIV-1 assay (Abbott Laboratories, Wiesbaden, Germany, limit of detection: 40 copies/mL) from 2013 until now.

#### 2.1.2. Genomic DNA Isolation and Single Genome Amplification (SGA) and Sequencing

A total of 1 × 10^7^ cryopreserved PBMCs were thawed, washed, and immediately after, the total genomic DNA was isolated with the addition of the DNAzol^®^ Reagent (Invitrogen, Waltham, MA, USA) under conditions recommended by the manufacturers. The isolated DNA was eluted in 100 µL DNase-free water and stored at −20 °C. SGA and the sequencing of DNA env sequences from PBMC were performed by limiting dilution nested PCR using conditions previously described [6]. The PCR products were sequenced using the ABI BigDye Terminator v3.1 Reaction Kit (Applied Biosystems, Foster City, CA, USA) in an ABI PRISM 3100 automated sequencer (Applied Biosystem). Chromatograms were assembled into contigs using the SeqMan Pro 11 software (DNASTAR Inc., Madison, WI, USA). Sequences resulting from chromatograms with double peaks or showing APOBEC3G/F mediated hypermutation as determined using Hypermut software [7] were discarded.

### 2.2. Sequence Analysis

DNA viral env sequences were aligned with HIV-1 subtype reference sequences using ClustalW and then manually edited, yielding a final alignment covering positions 7008–7650 relative to the HXB2 reference genome (Genbank accession number: K03455.1). Maximum-likelihood (ML) phylogenetic trees were reconstructed with the PhyML 3.0 program [8] using the most appropriate nucleotide substitution model (GTR + I + G) selected using program jModeltest v3.7 [9], the SPR branch swapping heuristic tree search algorithm, and the approximate likelihood-ratio test (aLRT) [10] for branch support. The genetic complexity of the proviral quasispecies was characterized by calculating the mean nucleotide diversity (π) using MEGA11 [11] as described previously [5,6], and the mean nucleotide divergence by performing a linear regression analysis of the root-to-tip distances against sampling time using the program TempEst [12]. The number of potential N-glycosylation sites (PNGSs) in env was predicted by N-GlycoSite (http://www.hiv.lanl.gov/content/sequence/GLYCOSITE/glycosite.html, accessed in 20 July 2022) [13].

### 2.3. Statistical Analysis

The Mann–Whitney U-test was used to compare the mean number of PNGSs between viral clades. The tests were two-sided, and *p* values ≤ 0.05 were considered as significant. Graphics and statistical analyses were performed using GraphPad v6 (Prism Software, Irvine, CA, USA).

### 2.4. Availability of Data

The sequences from the seven visits of subject EEC09 and the six visits of subject VC32 are part of a previous study and have been deposited in GenBank^®^ under accession numbers MH244562-MH244833. The HIV-1 Sequences generated during the current study (two EEC09 visits and three VC32 visits) were also deposited in GenBank^®^ under accession numbers OP169218 and OP169328.

## 3. Results

### 3.1. Clinical Aspects of Patients

Individuals EEC09 and VC32 have been followed up with HIV diagnoses in the early 2000s, and the longitudinal analysis of their plasma viremia and CD4+ T cell counts are displayed in Figure 1. Individual EEC09 showed undetectable plasma RNA viral load (<50–80 copies/mL) in most (21/24; 83%) measurements during the first 10 years of follow-up between January 2001 and December 2012. This was followed by two years of detectable plasma viremia in the low range (114–581 copies/mL) between 2013 and 2015 associated with a subtype F1 superinfection event, characterized in a previous study [5]. Individual EEC09 initiated ART in July 2015 and maintained undetectable plasma viremia (<50 copies/mL) until March 2020. Individual VC32 displayed a plasma RNA viral load in the low range (<50–230 copies/mL) from HIV-1 diagnosis in 2004 to 2013, after which the plasma viremia increased progressively up to 722 copies/mL in June 2015, which coincides with the detection of the subtype F1 superinfecting variant [5]. Individual VC32 refuses to initiate ART, and their plasma viremia gradually decreased and remained in the low range (45–156 copies/mL) between April 2016 and January 2021. In both individuals, the superinfecting subtype F1 virus was the dominant variant in the plasma compartment at the transient peak of the viremia. The EEC09 (595–2469 cells/µL) and VC32 (469–1005 cells/µL) subjects had CD4^+^ T lymphocyte counts within the normal range with no evidence of significant decline throughout follow-up (Figure 1).

### 3.2. Diversity and Evolution of DNA Viral Quasispecies Associated with PBMC

**Figure 2:**
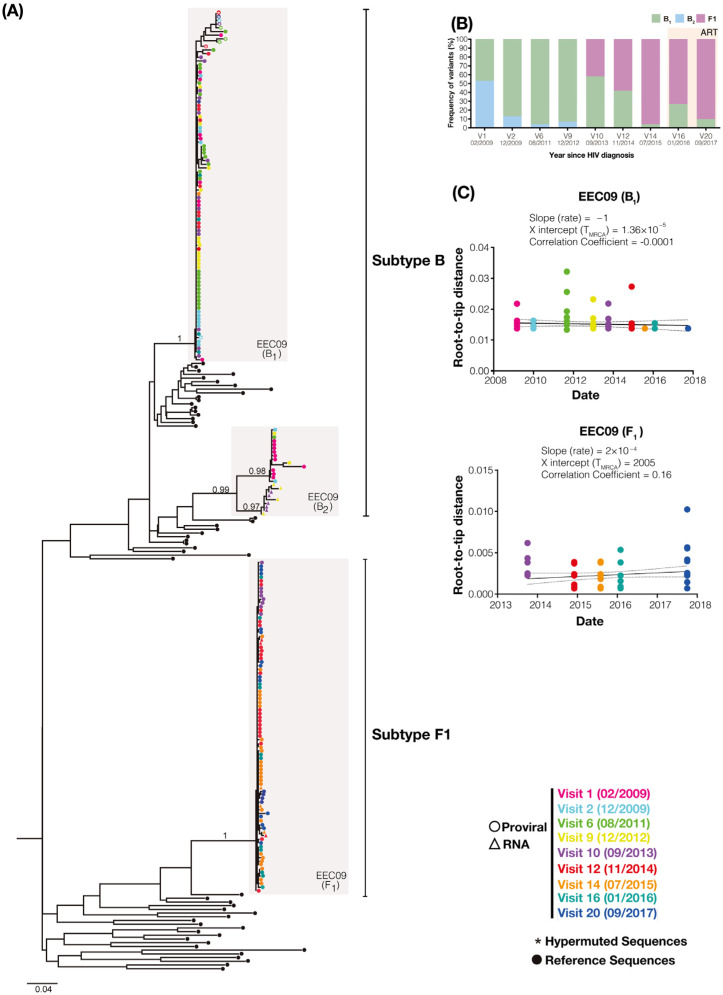
HIV-1 env gene sequences detected during follow-up of subject EEC09. (**A**) Longitudinal analysis of HIV-1 PBMC-associated DNA (circles) and plasma RNA (triangle) env sequences obtained from individual EEC09 between 2009 and 2017. Circles in the tips of the ML phylogenetic tree are colored according to the visit analyzed as shown in the legend at the bottom right. The gray shaded boxes highlight monophyletic clusters corresponding to each viral variant. White asterisks inside the circles highlight the sequences with APOBEC3G/F-mediated G to A hypermutations. Black circles point to the reference sequences, and vertical lines indicate subtype-specific clades (B and F1). Horizontal branch lengths are proportional to the bar at the bottom indicating nucleotide substitutions per site. The aLRT support is shown for key nodes. (**B**) The percentage of each viral variant at the PBMC compartment over time (years) is shown on the left Y-axis. (**C**) The plot of the root-to-tip distance against sequence sampling time of each viral variant. The colors of the circles represent the sampling time of DNA viral sequences according to the legend at the bottom right.

**Figure 3:**
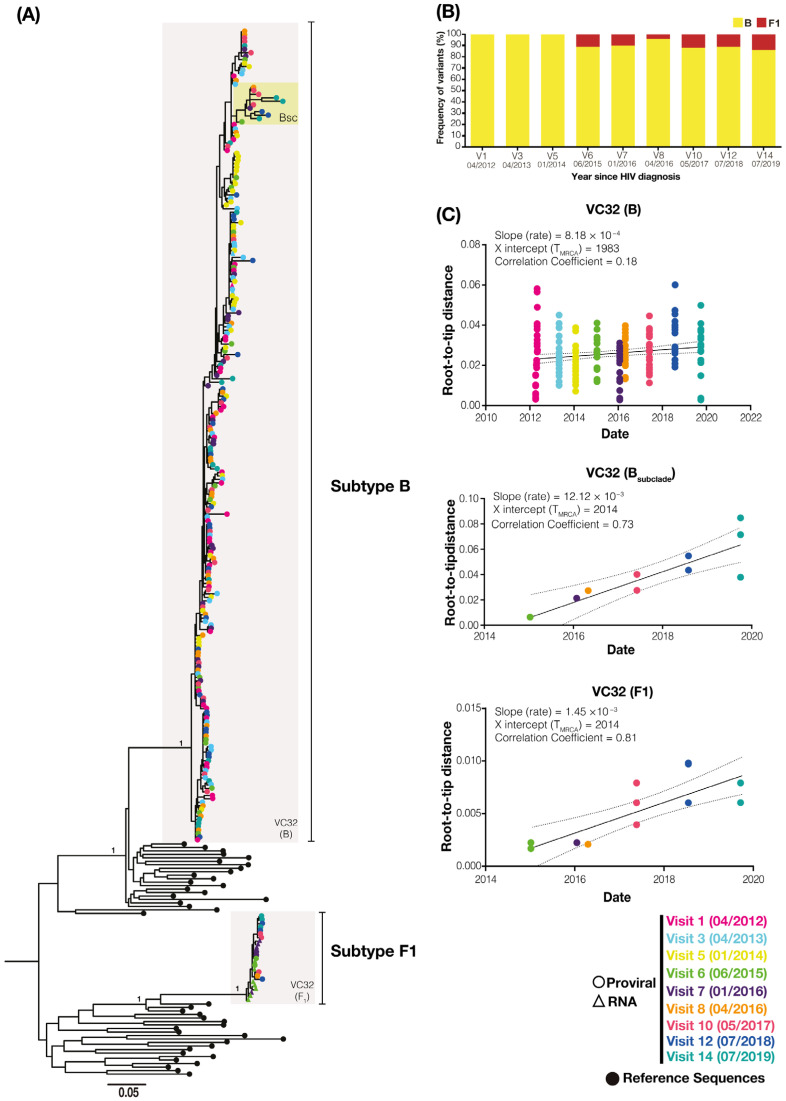
HIV-1 env gene sequences detected during follow-up of subject VC32. (**A**) Longitudinal analysis of HIV-1 PBMC-associated DNA (circles) and plasma RNA (triangle) env sequences obtained from individual VC32 between 2012 and 2019. Circles in the tips of the ML phylogenetic tree are colored according to the visit analyzed as shown in the legend at the bottom right. The shaded gray boxes highlight monophyletic clusters corresponding to each viral variant and the shaded yellow box indicates the monophyletic sub-cluster BSC. Black circles point to the reference sequences and vertical lines indicate subtype-specific clades (B and F1). Horizontal branch lengths are proportional to the bar at the bottom indicating nucleotide substitutions per site. The aLRT support is shown for key nodes. (**B**) The percentage of each viral variant at the PBMC compartment over time (years) is shown on the left Y-axis. (**C**) The plot of the root-to-tip distance against sequence sampling time of each viral variant. The colors of the circles represent the sampling time of DNA viral sequences according to the legend at the bottom right.

To better understand the long-term turnover dynamics of the primo-infecting and superinfecting variants, we performed limiting dilution env PCR to longitudinally assess the diversity of PBMC-associated viral quasispecies during two years of suppressive ART (between 2016 and 2017) in individual EEC09 and three years of spontaneous viremia control (between 2017 and 2019) in individual VC32. A total of 35 env sequences obtained from patient EEC09 between 2016 and 2017 were aligned with 170 env sequences previously recovered between 2009 and 2015. The ML phylogenetic analysis of env sequences of individual EEC09 confirms the triple infection previously described with two subtype B (hereafter called B1 and B2 variants) and one subtype F1 viruses (Figure 2A). The superinfecting subtype F1 virus was first detected in the DNA proviral in 2013 at a quite high frequency (42%) and became the dominant variant (>90%) before the initiation of ART in 2015 (Figure 2B). During the period of suppressive ART between 2016 and 2017, the B2 variant remained undetected, while the frequency of the B1 and F1 variants was very similar to that observed immediately before the ART initiation. We obtained 50 env sequences from patient VC32 between 2017 and 2019 that were aligned with 179 env sequences previously derived from samples collected between 2012 and 2016. The ML phylogenetic analysis of the env sequences of patient VC32 confirmed the dual infection with the subtypes B and F1 viruses previously described (Figure 3A). The superinfecting subtype F1 virus was first detected in the DNA proviral in 2015 at a low frequency (10%) and remained as a minor variant (<10%) at the DNA PBMC-associated compartment across all subsequent time points analyzed between 2016 and 2019 (Figure 3B). These findings demonstrate that primo-infecting and superinfecting variants persist in the DNA proviral of both HIV controllers for several years after superinfection and that their relative proportion remained stable under either fully suppressive ART or natural viremic control.

**Figure 4:**
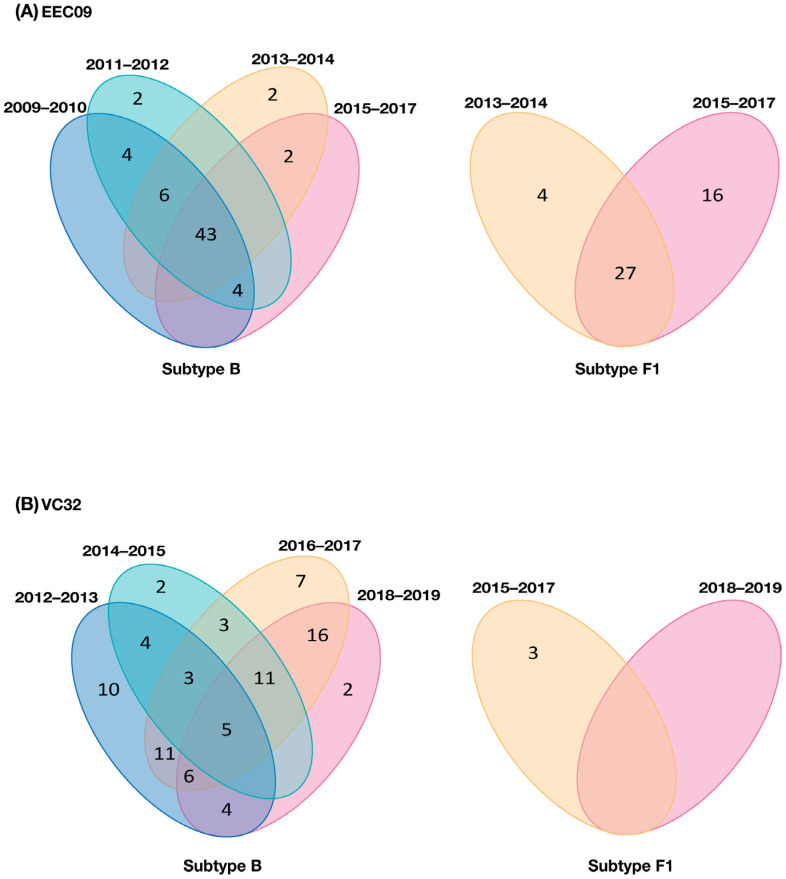
Number and temporal overlap of identical HIV-1 sequences (proviral clones) in subjects EEC09 (**A**) and VC32 (**B**). Venn diagrams contain the total number of identical proviral sequences, i.e., sequences that occurred more than once within the same participant, detected within subtypes B and F1 quasispecies at one (non-overlapping regions) or several (overlapping regions) time intervals during follow-up.

A closer inspection of DNA viral diversity and divergence, however, points to some differences in the underlying proviral dynamics over time in both subjects. Analysis of the root-to-tip distance against the sequence sampling time of each viral variant revealed no significant measurable increase in genetic divergence over time (*p* > 0.05) for the B1 or F1 DNA viral variants in subject EEC09 (Figure 2C). The evolutionary stasis of primo-infecting and superinfecting viruses in subject EEC09 coincides with a high frequency of identical DNA viral sequences, which could be used as a surrogate marker of “proviral clones”, defined here as identical proviral sequences that are likely a combination of infected cell clones maintained by clonal expansion and cells infected by the same or similar HIV strain. Of the 95 subtype B and 84 F1 subtype proviral sequences isolated from individual EEC09 over time, 63 (66%) and 47 (56%) sequences were classified as proviral clones, respectively, and a substantial fraction of subtype B (45%) and F1 (32%) proviral clones were detected across all the time points of follow-up (Figure 4A). In subject VC32, we found no evidence of significant divergence (*p* > 0.05) for the whole subtype B DNA proviral population. Still, we detect a significant divergence (*p* < 0.001) of a subtype B sub-clade (B_SC_) and of the subtype F1 DNA proviral population (Figure 3C). Interestingly, the B_SC_ and F1 variants were detected at the same time points, displayed correlation with the same X-intercept (2014), and were detected at roughly the same frequency (Figure 3C). Of the 227 subtype B proviral sequences detected in the VC32 individual, 84 (37%) were classified as proviral clones and a meager fraction (2%) corresponds to identical sequences detected across all the time points of follow-up (Figure 4B). Of the 12 subtype F1 sequences obtained, only three (25%) sequences detected in the period 2015–2017 were classified as proviral clones (Figure 4B). These findings support great stability and a very slow turnover rate of HIV-1-infected cells in subject EEC09 after ART initiation, but a majority renewal of proviruses in subject VC32 with the detectable divergence of the superinfecting proviral population and of a primo-infecting subpopulation during follow-up.

**Figure 5:**
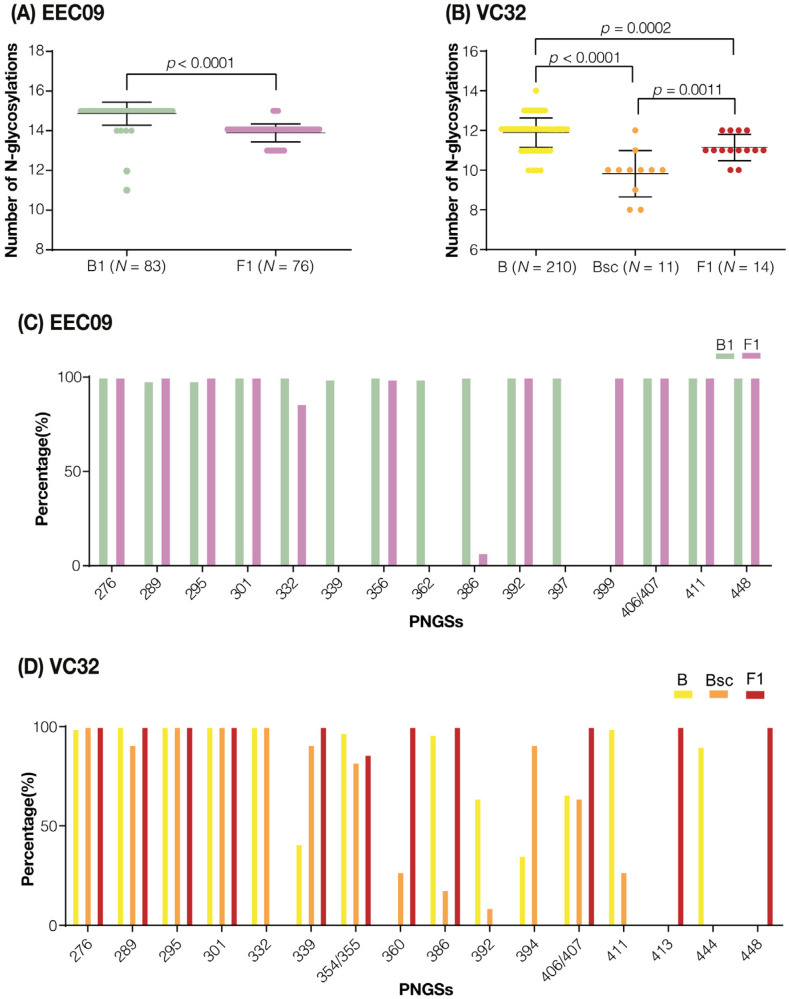
Analyses of N-linked glycosylation in the env gp120 C2-C4 region of viral sequences from subjects EEC09 and VC32. (**A**,**B**) Dot plots with the numbers of estimated PNGSs in env sequences from subtype B and F1 populations. Horizontal lines represent the mean and standard deviation. Two-tailed Mann–Whitney U tests were used. (**C**,**D**) Frequency of PNGSs in env sequences from subtype B and F1 populations. The X-axis represents the PNGSs identified in our study and the Y-axis shows the percentage frequency of the sequences from each variant found with PNGSs at the position. Positions in env gp120 are numbered according to the reference HIV-1 HXB2 strain.

The HIV-1 env glycan shield represents an essential mechanism of evasion from the humoral immune response [14]. While the mean number of env gp120 potential N-linked glycosylation sites (PNGSs) tends to be comparable between HIV-1 subtypes (~25 sites) and does not have a net increase over time at a population level [13], it typically increased at the intra-host level over the first years of chronic infection as HIV-1 adapts to host neutralizing antibodies (Nab) [15,16,17]. To investigate signs of humoral immune selection in proviral populations from primo-infecting and superinfecting variants, we compare the number of PNGSs within the C2–C4 region of env sequences over time. The mean number of PNGSs in the subtype B (14.9) and F1 (13.9) proviral populations from subject EEC09 were higher than in the subtype B (11.9), B_SC_ (9.8), and F1 (11.4) proviral populations from individual VC32 (Figure 5A,B). Within each subject, proviral populations of primo-infecting viruses displayed significantly (*p* < 0.0001) higher mean numbers of PNGSs than superinfecting viruses, with the notable exception of sub-clade B_SC_ that showed a significantly (*p* < 0.001) lower mean number of PNGSs than the other subject’s proviral populations. Overall, the PNGSs in the C2–V3 region were more conserved between the proviral populations than those in the C3–V4 region (Figure 5C,D). While all four PNGSs detected in the C2–V3 region (N276, N289, N295, and N301) were highly conserved in the viruses of both subjects, only 6/11 and 1/12 PNGSs in the C3–V4–C4 region were highly conserved among the proviral populations of subjects EEC09 and VC32, respectively. A comparison of the subtype B proviral populations in subject VC32 revealed several changes in the number of PNGSs in the C3–V4–C4 region, including the reduction in frequency or complete loss of some PNGSs (N386, N392, N411, and N444) and the increase in frequency or gain of others (N339, N360, and N394) in the evolving sub-clade B_SC_ in respect to the ancestral B population.

## 4. Discussion

Our findings revealed that primo-infecting and superinfecting variants persist in the DNA reservoir and that their relative proportion remained stable for several years after superinfection in both of the HICs here analyzed. The detection of both primo-infecting and superinfecting viruses in the HIV-infected cells for >3 years after superinfection in both HICs confirms the idea that proviral seeding occurs throughout the entire HIV infection course and that proviruses seeded may persist during fully suppressive or partial-suppressive viral replication [18,19,20,21,22]. Similar proviral dynamics were observed in a cohort of non-controllers from Kenya that initiated ART following superinfection and harbored both the initial and superinfecting viruses in the HIV-infected cells for >6 months of suppressive treatment [22]. The stable proportion of the primo-infecting and superinfecting variants in both HICs suggests that both fully suppressive ART or partial-suppressive natural control may create an environment that favors the slow turnover of the latent proviral of HIV-infected cells. However, the precise composition and proviral dynamics following superinfection were not the same in both HIC individuals.

In subject EEC09, there was an extensive reseeding of the proviral population immediately after superinfection, and its composition was skewed toward the more recently replicating superinfecting HIV variant. The ART, however, dramatically slowed the rate of the turnover of HIV-1-infected cells in subject EEC09 and stabilized the proviral population in the immediate pretherapy state, as has been previously observed in non-controllers [20,21] and VCs [23] on ART. Both primo-infecting and superinfecting viruses displayed no evidence of significant viral evolution (divergence) over time, and this evolutionary stasis of DNA viral populations coincides with the observation that a large fraction of both primo-infecting (66%) and superinfecting (56%) sequences were identical to at least another sequence of the viral quasispecies and most identical proviral sequences persisted during the entire follow-up. It was described that the long-term persistence of identical HIV proviral sequences could be driven by clonal expansion of HIV-infected memory CD4+ T cells [24,25,26,27,28,29,30]. These findings suggest that despite the significant turnover of the proviral population after the superinfection event in subject EEC09, the clonal expansion of long-lived HIV-infected cells was probably an important mechanism of HIV persistence in this subject before and after ART suppression. It is important to note that standard SGA may be insufficient to establish proviral clonality since identical sub-genomic HIV-1 sequences can result from either clonal expansion or from genetic bottlenecks that occurred with transmission or with the emergence of drug resistance mutation [31].

The proviral composition in subject VC32 remained skewed toward the primo-infecting HIV variant during the entire follow-up. It has been suggested that ART stabilizes the HIV reservoir by restoring the homeostatic transition from activated effector T cells to long-lived (CD127+ CD4+) T cells [32], and we may thus speculate that this individual was also able to preserve the CD4+ T cell homeostasis following superinfection. Our analyses of the landscape of identical sequences, however, revealed that the overall fraction of clonal proviruses of primo-infecting (37%) and superinfecting (25%) variants in subject VC32 was much lower than in subject EEC09, and few of them persisted over the entire follow-up. This indicates that despite the overall stability in the relative proportion of primo-infecting and superinfecting viruses observed in subject VC32 during follow-up, the persistence of both viral variants in this subject seems to be mainly driven by the continuous reseeding of the proviral population rather than by the clonal expansion of long-lived HIV-infected cells. Of note, such proviral reseeding was associated with a significant divergence of the subtype F1 and a subpopulation of the subtype B virus, which may reflect some level of residual replication of both primo-infecting and superinfecting variants over time. Whether such divergence is a transient phenomenon or may reflect an early signal of the potential risk of a future virologic breakthrough by the primo-infecting and/or superinfecting variants in this patient is currently unknown.

One interesting finding was the detection of the B_SC_ subclade that comprises a minor fraction (6%) of all primo-infecting subtype B sequences recovered from subject VC32. Similar to the superinfecting subtype F1 virus population, the B_SC_ subclade comprises sequences from 2015–2019 and displayed a significant divergence over time. More remarkably, the X-intercept of the correlation of divergence (root-to-tip distance) against the sequence sampling time, which could be used as a proxy of the time of the most recent common ancestor (TMRCA), was traced to 2014 for both the subtype F1 and B_SC_ variants, coinciding with the time interval of the superinfection event (January 2014–June 2015). This evidence clearly supports that the B_SC_ subclade probably arose and started to diverge around the time of the subtype F1 superinfection. One hypothesis to explain such coincidences is that the B_SC_ subclade actually represents a BF1 variant generated by recombination between primo-infecting and superinfecting viruses immediately after superinfection. HIV-1 intersubtype recombination following superinfection has been previously described in several non-controllers’ individuals with variable outcomes of HIV-1-infected cell reseeding [22,33,34]. Of note, the subtype F1 and B_SC_ variants combined comprise a minor fraction (<30%) of the quasispecies population at all time points, supporting the conclusion that the proviral population in subject VC32 remained skewed toward the primo-infecting HIV variant during the entire follow-up.

Previous studies support that the numbers of PNGSs in env sequences may vary significantly between patients and even between cell compartments of a single individual according to the level of active replication, consistent with the notion that viral quasispecies with a more restricted replication and evolution displayed a lower mean number of PNGSs in env and thus an overall lower resistance to NAb responses [35,36]. Our analyses, however, revealed that the mean numbers of PNGSs in env sequences of replicative subtype F1 superinfecting proviruses were significantly lower than in sequences from non-evolving subtype B primo-infecting proviruses. This apparent inconsistency could be explained by the quite different time periods of intra-host evolution between primo-infecting and superinfecting viruses. The number of PNGSs in env typically increased over the first years of infection, as the infecting virus adapts to the autologous Nab [16,17,37]. Thus, the differences observed may have resulted from the longer time of intra-host persistence of the primo-infecting subtype B virus in the face of an evolving repertoire of autologous Nab and the much lower humoral immune pressure against the superinfecting subtype F1 virus immediately after superinfection.

One limitation of our study is that we do not test for the presence of autologous NAb against primo-infecting and superinfecting viruses over time, making it difficult to obtain solid conclusions about the potential impact of changes at PNGSs on patient control in vivo. We hypothesize that the high mean number of PNGSs detected in env sequences of primo-infecting subtype B viruses may have resulted from a mechanism of ongoing viral escape from autologous NAb as observed in typical progressors [16,17,38]. Although the evolving glycan shield may have contributed to HIV-1 persistence in subjects EEC09 or VC32, it was not associated with virologic breakthroughs previous to superinfection in those HICs. Meanwhile, the relatively low number of PNGS detected in env subtype F1 sequences may reflect the short period of intra-host evolution and the lower titers of autologous NAb raised against the superinfecting viral variant in the first months after superinfection, which coincides with higher levels of viral replication of superinfecting than primo-infecting viruses. As the subjects developed autologous NAb against the superinfecting viruses, we may expect that the mean number of PNGS in env subtype F1 sequences increase over time and the viremia returns to levels observed before superinfection. Unfortunately, we could not test this prediction because subject EEC09 initiated ART shortly after superinfection and the number of subtype F1 clones recovered from subject VC32 at each visit was very low (*n* < 5).

Our analysis also revealed that the mean numbers of PNGSs in env sequences of the evolving B_SC_ subpopulation (~10) of subject VC32 were significantly lower than that observed in the non-evolving B ancestral population (~12). One hypothesis to explain such a reduction may be that the B_SC_ variant evolved from an inactive cell compartment that comprised a B ancestral subpopulation with a relatively low number of PNGSs. Indeed, we identified that a low fraction (7.7%) of the B ancestral population carried low numbers of PNGSs (*n* ≤ 10), comparable to those observed in the B_SC_ subpopulation. Our analysis, however, also revealed the gain and loss of some PNGSs in the B_SC_ subpopulation with respect to the B ancestral population, supporting that the glycan shield of the B_SC_ subpopulation may have been evolving since its emergence. Interestingly, the major difference resulted from a reduction in the number of PNGSs at the V4–C4 region of B_SC_ with respect to the B ancestral populations, and a previous study demonstrated that the loss of glycosylation in the V4–C4 region may have a significant increase in viral infectivity [39]. The overall lower number of PNGSs in the evolving B_SC_ subpopulation with respect to the non-evolving one might thus represent a signature of increased viral infectivity, rather than of lower resistance autologous to NAb.

## 5. Conclusions

In summary, the longitudinal follow-up of HIV DNA quasispecies in two superinfected HICs confirms the notion that the HIV-1 proviral dynamics in untreated HICs are shaped by both the long-term persistence of HIV-infected cells and continuous reseeding. Our findings demonstrate that the relative abundance of initial and superinfecting viral strains remained mostly unchanged for several years under either suppressive ART or persistent low-level (<200 copies/mL) viremia. However, the fully suppressive treatment seems to be more efficient in blocking the proviral reseeding and favoring the long-term persistence of HIV-infected cells than partial-suppressive natural immunity. Long-term follow-up should be necessary to assess whether such differences could be associated with a differential risk of future virologic breakthroughs over time. Furthermore, full-length virus sequencing will be also crucial to assess the predominance of primo-infecting, superinfecting, and potential recombinant variants over time.

## Data Availability

The GenBank database accession numbers for the HIV-1 env sequences described in this study were deposited in GenBank^®^ under accession numbers OP169218 and OP169328.
